# Does Ketogenic Diet Used in Pregnancy Affect the Nervous System Development in Offspring?—FTIR Microspectroscopy Study

**DOI:** 10.1021/acschemneuro.3c00331

**Published:** 2023-07-20

**Authors:** Marzena Rugiel, Zuzanna Setkowicz-Janeczko, Wojciech Kosiek, Zuzanna Rauk, Kamil Kawon, Joanna Chwiej

**Affiliations:** † Faculty of Physics and Applied Computer Science, AGH University of Krakow, Krakow 30-059, Poland; ‡ Institute of Zoology and Biomedical Research, Jagiellonian University, Krakow 31-007, Poland

**Keywords:** ketogenic diet, pregnancy, brain development, Fourier transform infrared microspectroscopy, biochemical analysis, principal component analysis (PCA)

## Abstract

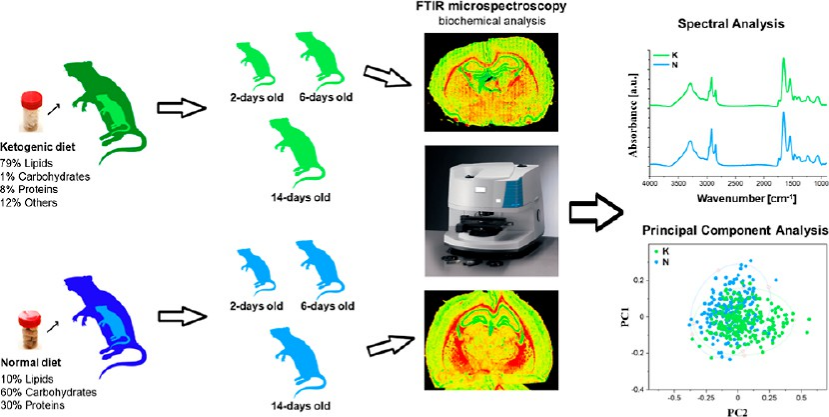

Anti-seizure medications used during pregnancy may have transient or long-lasting impact on the nervous system of the offspring. Therefore, there is a great need to search for alternative therapies for pregnant women suffering from seizures. One of the solutions may be the use of the ketogenic diet (KD), which has been successfully applied as a treatment of drug-resistant epilepsy in children and adults. However, the risks associated with the use of this dietary therapy during pregnancy are unknown and more investigation in this area is needed. To shed some light on this problem, we attempted to determine the potential abnormalities in brain biomolecular composition that may occur in the offspring after the prenatal exposure to KD. To achieve this, the female Wistar rats were, during pregnancy, fed with either ketogenic or standard laboratory diet, and for further studies, their male offspring at 2, 6, or 14 days of age were used. Fourier transform infrared microspectroscopy was applied for topographic and quantitative analysis of main biological macromolecules (proteins, lipids, compounds containing phosphate and carbonyl groups, and cholesterol) in brain samples. Performed chemical mapping and further semi-quantitative and statistical analysis showed that the use of the KD during pregnancy, in general, does not lead to the brain biochemical anomalies in 2 and 6 days old rats. The exception from this rule was increased relative (comparing to proteins) content of compounds containing phosphate groups in white matter and cortex of 2 days old rats exposed prenatally to KD. Greater number of abnormalities was found in brains of the 14 days old offspring of KD-fed mothers. They included the increase of the relative level of compounds containing carbonyl groups (in cortex as well as multiform and molecular cells of the hippocampal formation) as well as the decrease of the relative content of lipids and their structural changes (in white matter). What is more, the surface of the internal capsule (structure of the white matter) determined for this age group was smaller in animals subjected to prenatal KD exposure. The observed changes seem to arise from the elevated exposition to ketone bodies during a fetus life and the disturbance of lipid metabolism after prenatal exposure to the KD. These changes may be also associated with the processes of compensation of mother organism, which slowly began to make up for the deficiencies in carbohydrates postpartum.

## Introduction

Treatment of patients with epilepsy during pregnancy is challenging because most of the available anticonvulsant drugs are teratogenic. Their use in critical stages of fetal development may lead to transient or long-term side effects in the nervous system of the offspring that involve anatomical and behavioral anomalies.^1,2^ Because of the fact that antiepileptic medicines that are safe for pregnant women have not been found so far, the search for new pharmacological agents and alternative therapies that will both benefit the mother and be safe for the offspring is ongoing. One of the possibilities may be the utilization of a ketogenic diet (KD), which has been successfully used in the treatment of various type of epilepsy (including drug-resistant epilepsy) in infants, children, and adults.^3−7^

KD is a high-fat and low-carbohydrate diet that goal is to imitate a beneficial effects of fasting but without depriving the organism calories demanded to metabolism.^4,8^ During KD, fat intake should be around 80% of total caloric consumption, thus changing the main energy source for metabolism from carbohydrates to fats and inducing ketone body production in the liver.^4,9,10^ Acetoacetate, which spontaneously converts into acetone, and β-hydroxybutyrate are the ketone bodies (KBs) produced from fatty acids in the liver which during KD become the major source of energy for the central nervous system, replacing glucose.^4,10^ KBs are able to cross the blood–brain barrier through monocarboxylate transporters (MCTs) of endothelial cells and astroglia.^11^ However, the mechanism of KD action in patients with drug-resistant epilepsy is not fully understood.^12^ The diet is supposed to act on many levels of nerve cells function, including the influence on the neuronal metabolism and excitability.^7^ Its effectiveness in reducing the frequency of epileptic episodes may be associated with the increased availability of ketone bodies, the elevated concentration of free fatty acids and a decreased content of glucose in the blood and cerebrospinal fluid, which may have an anticonvulsant effect.^6,7^ Other studies pointed out that KB also exert a direct inhibitory effect on the vesicular glutamate transport^13^ and have an influence on neurotransmitter release and ATP sensitive potassium channels.^14^

There is a lack of medical data on the risks of using KD during pregnancy in humans.^15^ According to our best knowledge, there are only two publications documenting the pregnancy of women treated with KD.^16,17^ In the first article, a case of 19 year-old woman with glucose transporter type 1 deficiency syndrome (Glut1DS) treated with KD before and during gestation was presented.^16^ Her child, also fed with KD as a neonate, was monitored for the next 5 years later and described as an asymptomatic and excelling developmentally.^16^ The second article included two case reports using KD therapy for epilepsy during pregnancy.^17^ The authors of the cited paper claim that non-pharmacological epilepsy therapies, including KD, may be effective during pregnancy. However, they also highlight the necessity of further patient and offspring monitoring to identify potential long term side effects of the dietary therapies.^17^ The results of research based on the animal models have shown that the prenatal exposure to KD may cause the disruptions in the embryotic organ growth of rodents, lead to neuroanatomical changes and influence the behavior of offspring later in life.^18−20^ According to Sussman et al. the brain is the organ especially susceptible to the changes influenced by KD and the possible anomalies include the relative reduction of the cortex, hippocampus, corpus callosum, fimbria, and the lateral ventricles and a relative increase of the hypothalamus and medulla volume.^20^ As reported by the mentioned study, the observed abnormalities may be the consequence of certain brain regions preferences for ketones utilization during prenatal development and increased efficiency of energy production from them.^20^ KD is also associated with the reduced protein intake. The lack of these macromolecules during pregnancy in rats reveal in persistent or reversible anatomical and functional changes to the brain of their pups, and may lead to the delay in the appearance of reflexes.^21,22^ There is also a number of studies showing that a mother nutrition during pregnancy, especially high-fat diet, may affect the development of the progeny.^23−25^ The numerous papers report negative effects of the exposure to such a diet at the prenatal stage.^26−30^ However, there is also study suggesting that in utero exposure to high-fat diet may play a protective role for offspring brain health later in the adulthood.^31^

The methods of vibrational spectroscopy have not been used so far to image biochemical changes occurring in the brain as a result of prenatal exposure to KD in any of the animal models. In the present study, due to its many advantages, Fourier transform infrared (FTIR) microspectroscopy was applied. The method allows to obtain high quality molecular spectra (high signal to noise ratio and good spectral resolution) of various biological systems.^32^ Because of high speed of data acquisition, it is possible to examine relatively large areas of samples and/or increase the statistics of examined cases what is very important in biomedical research. Another benefit of FTIR microspetroscopy is the possibility of utilization, depending on the type of examined sample, of different measurement modes (transmission, transflection, and attenuated total reflection).^32^ Thanks to the combination with optical microscopy, FTIR microspectroscopy allows to identify microscopic details in the sample, simultaneously providing the information concerning the presence of particular functional groups, bonding types or molecular conformations in the examined area.^32−34^ What is more, it is non-invasive method and require only small amounts of material and minimum sample preparation.^32−34^

In our research, FTIR microspectroscopy was applied to identify the potential anomalies in the content and structure of main biological macromolecules that occur in the brain of the progeny of rats fed with KD during pregnancy. The levels of the biomolecules such as proteins, lipids, compounds containing phosphate and carbonyl groups, cholesterol, and its esters were included in the study. What is more, the changes in the structure of proteins and lipids were analyzed. In the study, we were focused on the period of intensive brain postnatal development and, therefore, the animals at the age of 2, 6, and 14 days old were included in the experiment.

## Results

To answer the question whether prenatal exposition to KD modifies the content and structure of biological macromolecules within the white matter, brain cortex, and hippocampal cellular layers, the biochemical composition of these areas was compared for offspring of females fed during pregnancy with ketogenic (K group) and normal (N group) diet. The performed comparisons included the qualitative analysis of chemical maps presenting the distribution of the absorption bands characteristic for the main biomolecules, as well as the evaluation of the spectral differences between the examined animal populations. Moreover, the semi-quantitative biochemical analysis based on absolute and relative intensities of selected absorption bands was done and statistical relevance of the observed differences between experimental groups and appropriate controls was verified with the Mann–Whitney *U* test.

### Chemical Mapping

#### Distribution of Proteins and Structural Changes of Proteins and Lipids

**Figure 1:**
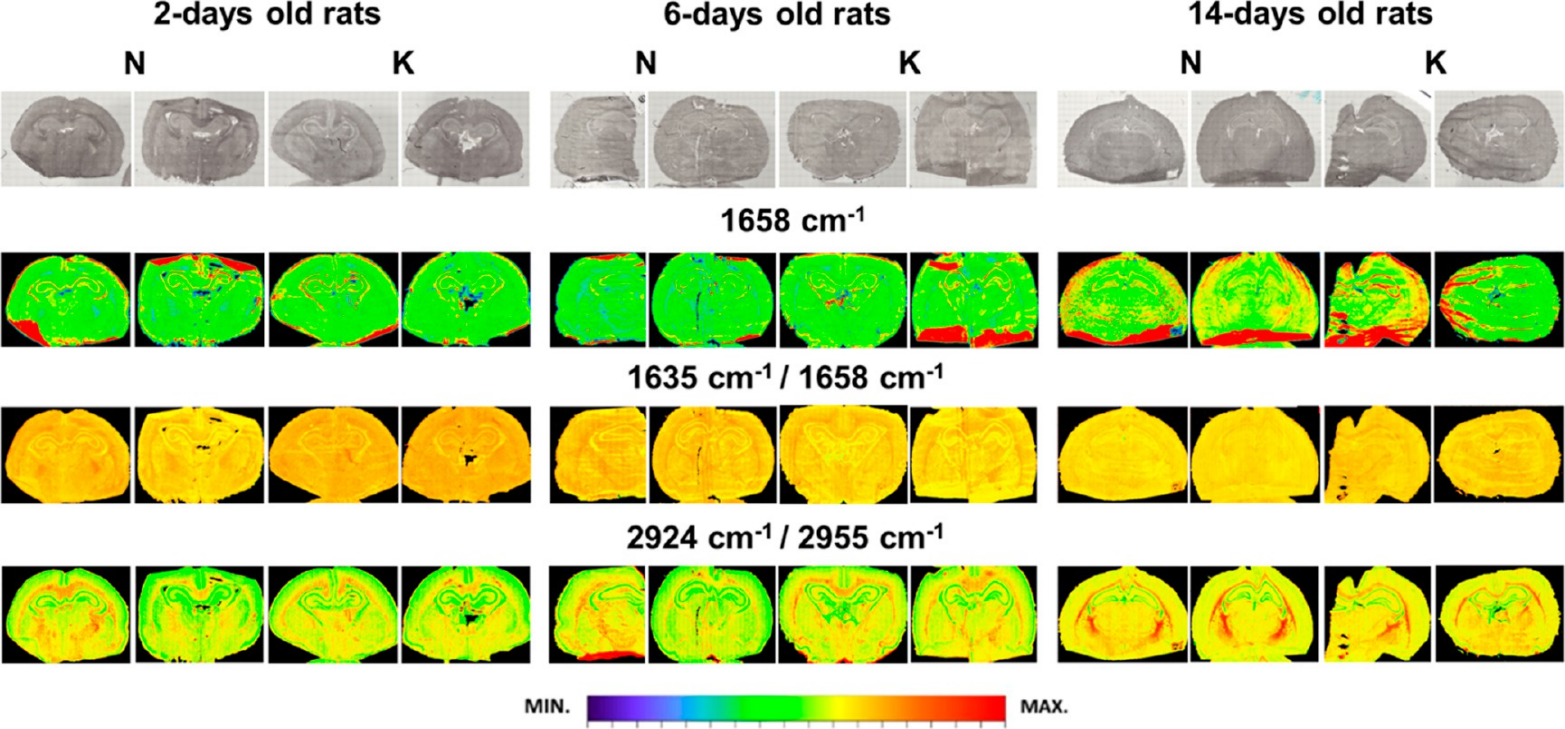
Exemplary chemical maps presenting the distributions of the amide I band as well as the ratios of absorbance at the wavenumbers of 1635 and 1658 cm^–1^ and 2924 and 2955 cm^–1^, obtained for the brain samples taken from 2, 6, and 14 days old rats, which during prenatal life were exposed to the ketogenic (K) and normal (N) diets. Additionally, in the first row, the microscopic views of the scanned tissue areas are shown. For better visualization of anatomical details in brain sections, the reader is referred to Figures S1–S3 of the Supporting Information.

As one can see from Figure 1, the chemical maps presenting the distribution of the amide I band intensity (1658 cm^–1^) within examined brain slices did not show the differences in proteins accumulation between the rats from K and N groups at any stages of their postnatal development. Therefore, in the further study, the intensity of this band was applied as normalizing parameter when relative content of examined biomolecules in the tissue was calculated.

Also, for the ratio of the absorbance at the wavenumbers of 1635 and 1658 cm^–1^, which is used to identify the changes in the relative secondary structure of proteins, none differences between experimental and control groups were noticed. The chemical maps imaged the ratio of the intensity of the bands at the wavenumbers of 2924 and 2955 cm^–1^ did not point at the existence of the anomalies in the structure of lipids in the 2 and 6 days old offspring of females fed with KD. For 14 days old rats, however, some differences in the surface of brain areas characterized by an elevated ratio of these lipid bands intensity were found. As one can see from Figure 1, the mentioned region is localized under the hippocampal formation and corresponds to the area of internal capsule and its surface is noticeably larger for animals from N comparing to K group.

The developmental changes observed in the offspring of females fed with ketogenic and standard diets were similar and included an increase in the intensity of the amide I band and the ratio of the examined lipid bands between the 6th and 14th days of rat postnatal life.

#### Distribution of Lipids, Cholesterol and Its Esters

**Figure 2:**
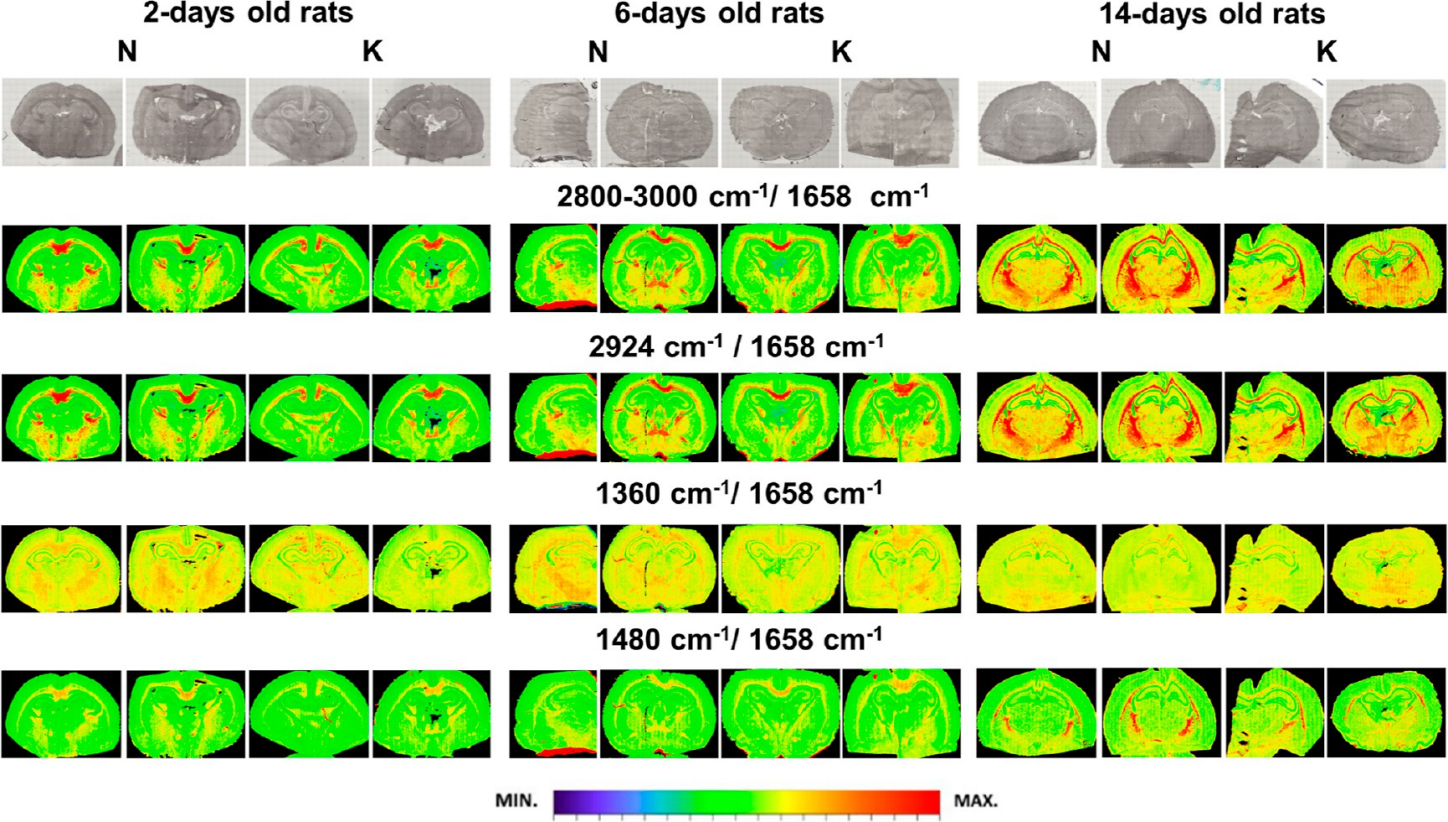
Exemplary chemical maps presenting the distributions of the relative (comparing to amide I band) intensity of selected absorption bands (2924, 1360, and 1480 cm^–1^) and the lipid massif (2800–3000 cm^–1^) for brain samples taken from 2, 6, and 14 days old rats, which during prenatal life were exposed to the ketogenic (K) and normal (N) diets. Additionally, in the first row, the microscopic views of the scanned tissue areas are shown.

The chemical maps obtained for 2 and 6 days old rats (Figure 2) did not show any general differences in the relative content of lipids (2800–3000/1658 and 2924/1658 cm^–1^) as well as cholesterol and its esters (1360/1658 and 1480/1658 cm^–1^) between the experimental groups and appropriate controls. However, for 14 days old rats, the area of internal capsule was significantly smaller for experimental group comparing to the control one and characterized by lower values of parameters describing the relative content of lipids as well as cholesterol and its esters. Moreover, as it can be seen in Figure 2, the relative content of the mentioned biomolecules was increasing significantly between 6th and 14th day of postnatal brain development independently on the mother diet.

#### Accumulation of Compounds Containing Phosphate and Carbonyl Groups

**Figure 3:**
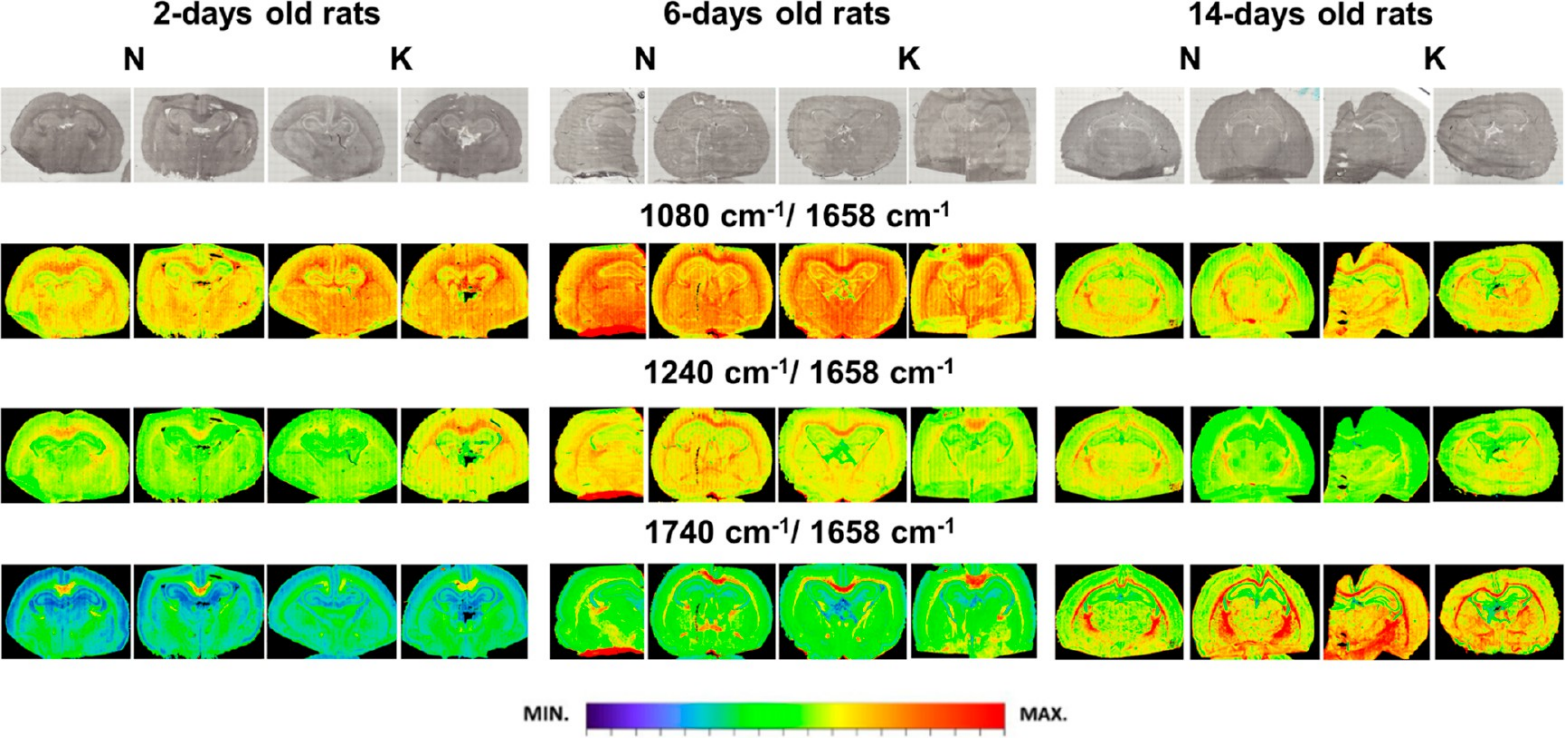
Exemplary chemical maps presenting the distributions of the relative (comparing to amide I band) intensity of 1080, 1240, and 1740 cm^–1^ absorption bands for brain samples taken from 2, 6, and 14 days old rats, which during prenatal life were exposed to the ketogenic (K) and normal (N) diets. Additionally, in the first row, the microscopic views of the scanned tissue areas are shown.

As one can see from the Figure 3, the general increase in the ratio of bands 1080/1658 cm^–1^ was found for 2 days old rats exposed in prenatal life to the KD. The similar relation was observed for the relative intensity of the band 1740 cm^–1^ originating from compounds containing carbonyl groups, but only in the case of 14 days old rats and, especially, for the area of the brain cortex.

Taking into account the stage of brain development, independently on the animal group (N or K), the highest relative intensity of the bands at 1080 and 1240 cm^–1^ was noticed for 6 days old rats. In turn, the intensity ratio of bands at 1740 and 1658 cm^–1^ showed gradual increase from 2nd until the 14th day of postnatal life.

#### Principal Component Analysis

**Figure 4:**
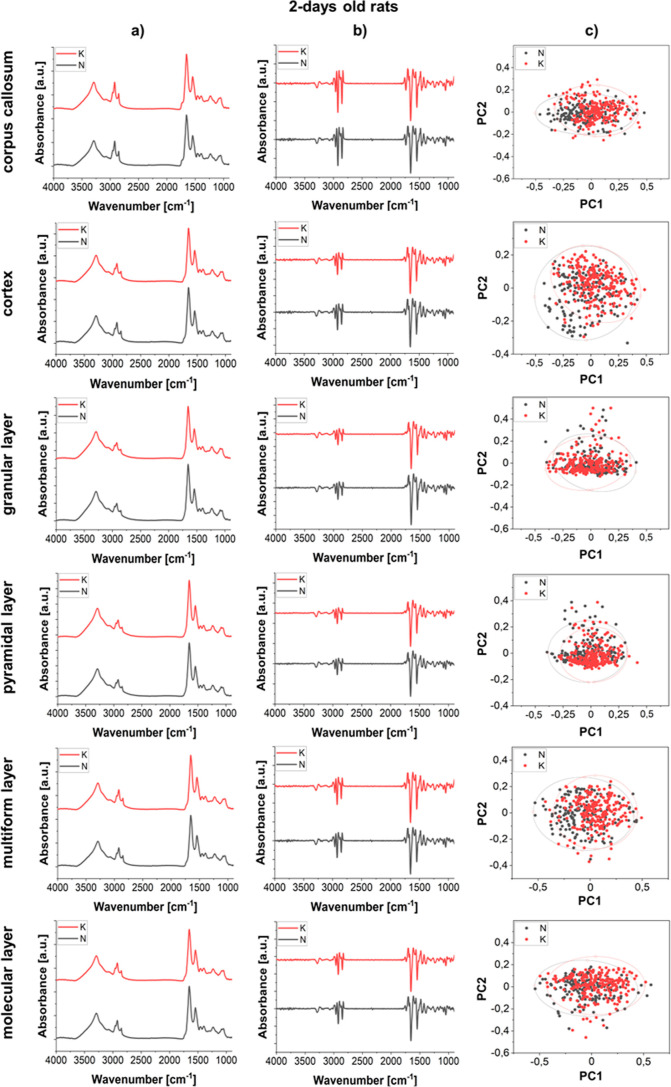
Comparison of the average spectra (column a) and the second derivative spectra (column b) obtained for corpus callosum, cerebral cortex, and hippocampal cellular layers (granular, pyramidal, multiform, and molecular) for 2 days old offspring of females fed with the KD (red) and standard laboratory diet (black). The results of PCA done on the second derivatives of vector-normalized spectra are shown in the column c.

**Figure 5:**
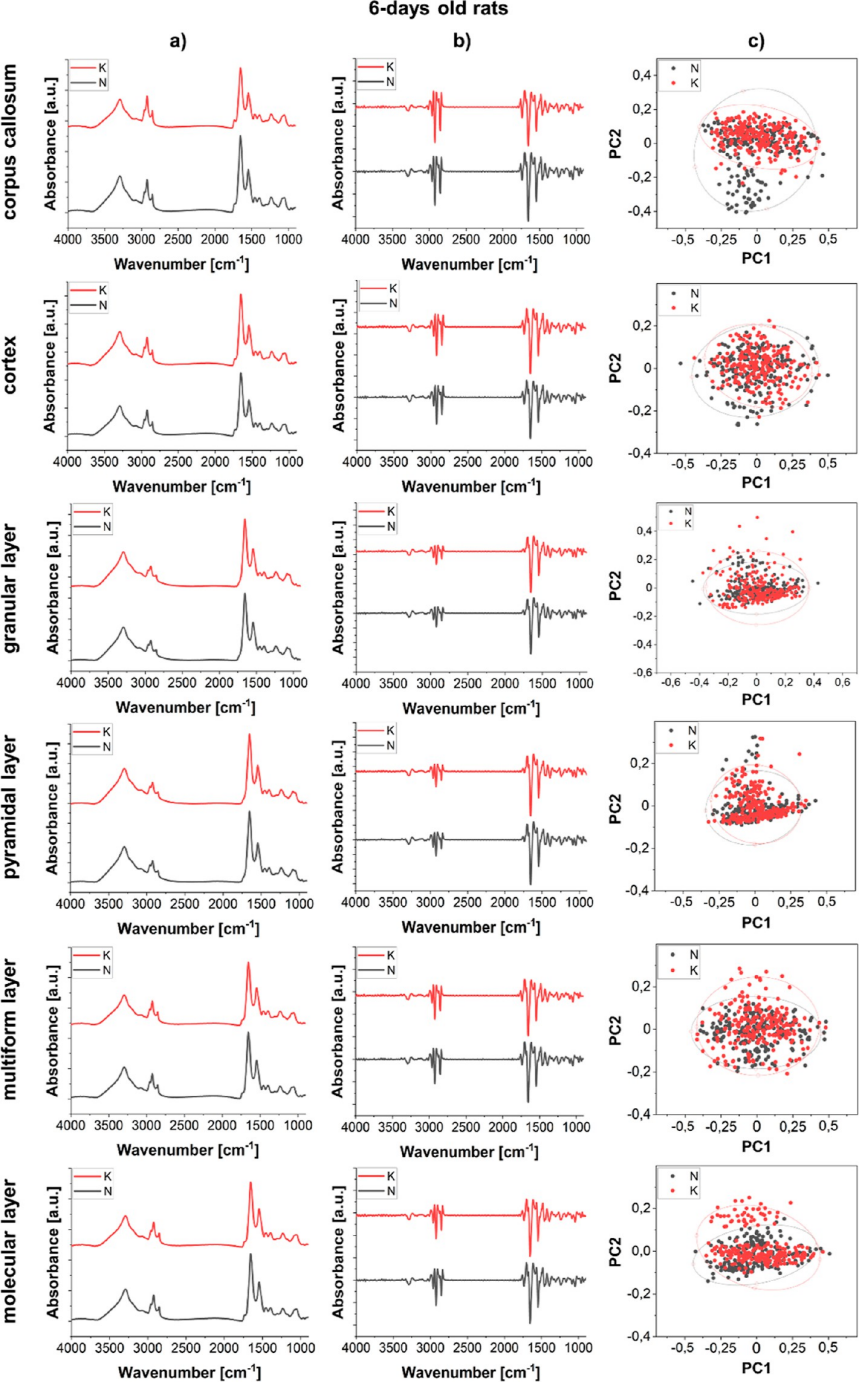
Comparison of the average spectra (column a) and the second derivative spectra (column b) obtained for corpus callosum, cerebral cortex, and hippocampal cellular layers (granular, pyramidal, multiform, and molecular) for 6 days old offspring of females fed with the KD (red) and standard laboratory diet (black). The results of PCA done on the second derivatives of vector-normalized spectra are shown in the column c.

**Figure 6:**
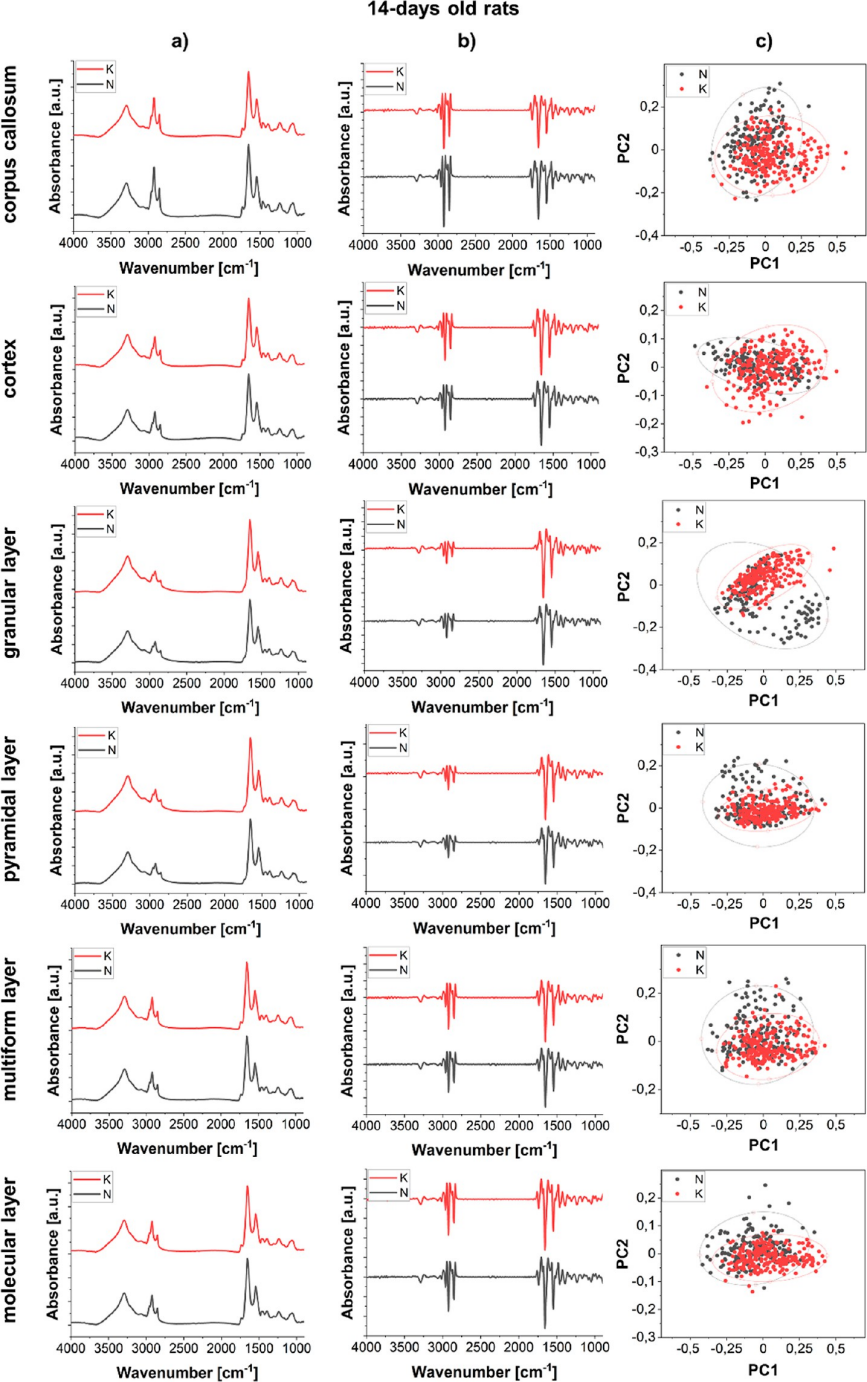
Comparison of the average spectra (column a) and the second derivative spectra (column b) obtained for corpus callosum, cerebral cortex, and hippocampal cellular layers (granular, pyramidal, multiform, and molecular) for 14 days old offspring of females fed with the KD (red) and standard laboratory diet (black). The results of PCA done on the second derivatives of vector-normalized spectra are shown in the column c.

In order to check potential biochemical abnormalities which may appear in the offspring of KD-fed females, the recorded spectral data were subjected to PCA. This advanced statistical method was used, separately, for IR spectra measured in the area of corpus callosum and brain cortex as well as in four cellular layers of hippocampal formation (granular, pyramidal, multiform, and molecular layer). The average spectra, their second derivatives and the results of PCA carried out on the second derivative spectra subjected to vector normalization are presented in the Figures 4, 5, and 6 for the 2, 6, and 14 days old rats, respectively. The performed PCA showed, for all examined brain regions and cellular layers as well as any stages of postnatal development, that the absorption spectra recorded for animals from experimental and control groups did not differ significantly. As the analysis took into account all the components of the collected IR spectra, its results suggest that there are no global quantitative biomolecular differences between the offspring of females fed during pregnancy with ketogenic and normal diets.

#### Mann–Whitney *U* Test

**Figure 7:**
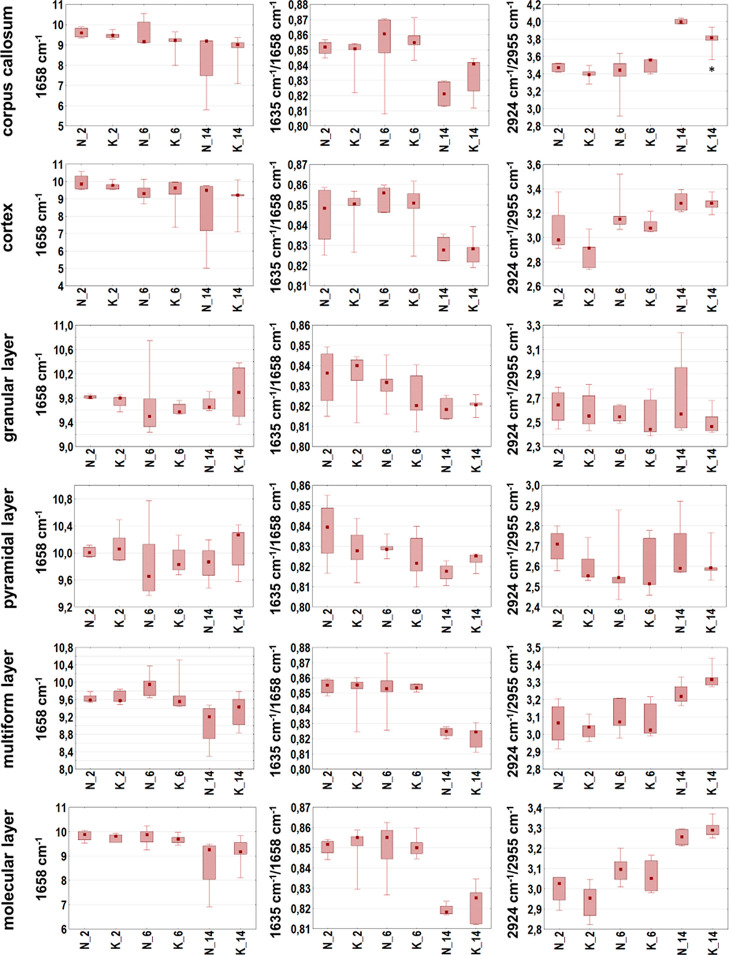
Box-and-whisker plots presenting the spread of the biochemical parameters values (integrated band areas or their ratios) in corpus callosum, cortex, and four hippocampal layers (granular, pyramidal, multiform, and molecular) for experimental and control rats (K and N groups, respectively) at examined stages of postnatal development (2, 6, and 14 days of life). Statistically significant differences (Mann–Whitney *U* test, 95% confidence level) between experimental groups and appropriate controls were marked with *.

**Figure 8:**
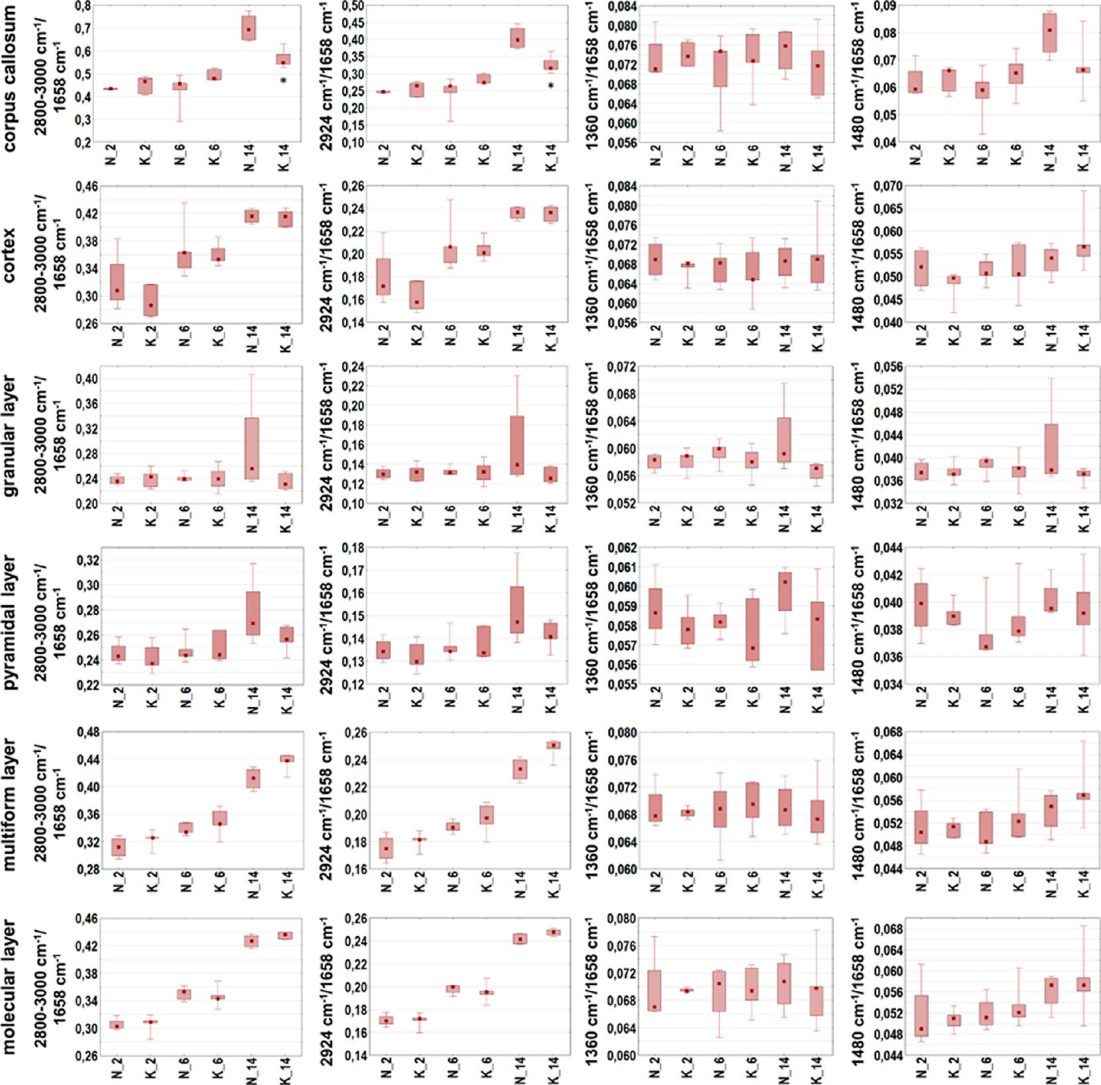
Box-and-whisker plots presenting the spread of the biochemical parameters values (integrated band areas or their ratios) in corpus callosum, cortex, and four hippocampal layers (granular, pyramidal, multiform, and molecular) for experimental and control rats (K and N groups, respectively) at examined stages of postnatal development (2, 6, and 14 days of life). Statistically significant differences (Mann–Whitney *U* test, 95% confidence level) between experimental groups and appropriate controls were marked with *.

**Figure 9:**
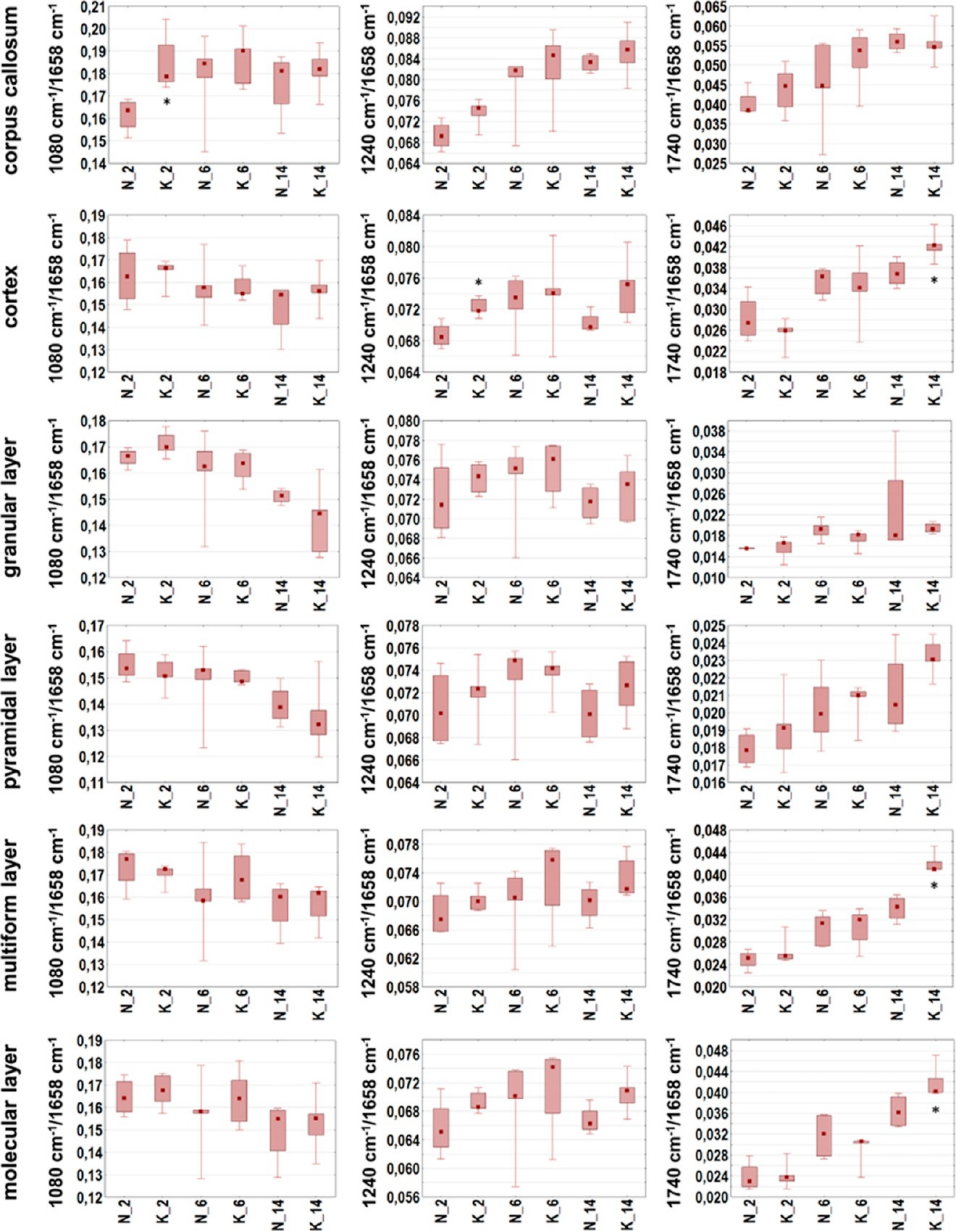
Box-and-whisker plots presenting the spread of the biochemical parameters values (integrated band areas or their ratios) in corpus callosum, cortex, and four hippocampal layers (granular, pyramidal, multiform, and molecular) for experimental and control rats (K and N groups, respectively) at examined stages of postnatal development (2, 6, and 14 days of life). Statistically significant differences (Mann–Whitney *U* test, 95% confidence level) between experimental groups and appropriate controls were marked with *.

The next step of the study was semi-quantitative analysis of the spectral data. For each animal, the absolute or relative mean intensities of the chosen absorption bands (Table 3) in the areas/cellular layers of interest were calculated. The intensities were calculated as integrated peak areas. The obtained results are compared in Figures 7–9, presenting the minimum and maximum values as well as medians of the biochemical parameters determined for particular experimental groups (K_2, K_6, and K_14) and appropriate controls (N_2, N_6, and N_14).

To verify if KD used during prenatal life modifies the content and structure of biomolecules within the brains of the offspring, the semi-quantitative data were subjected to the statistical analysis with the use of the Mann–Whitney *U* test. The statistically significant differences found between experimental groups and controls were marked with stars in Figures 7–9.

None statistically relevant differences in the biomolecular composition of examined areas/cellular layers were found for the group of 6 days old rats. Such differences, however, were detected in the part of white matter called corpus callosum in the case of 14 days old animals fed prenatally with KD. As one can see in Figures 7 and 8, they included the diminished relative intensities of lipid bands (2800–3000/1658 and 2924/1658 cm^–1^) and structural changes of these biomolecules (2924/2955 cm^–1^). For the same age group, higher relative content of compounds containing carbonyl groups (1740/1658 cm^–1^) in cortex and two cellular layers of hippocampus (multiform and molecular) was also found (Figure 9), what confirmed the prior results of the topographic biomolecular analysis. As it can be seen in Figure 9, the performed statistical analysis allowed also to find the differences in the accumulation of biomolecules between 2 days old offspring of females fed with ketogenic and normal diet. The rats representing the experimental group showed higher relative content of compounds containing phosphate groups in corpus callosum (1080/1658 cm^–1^) and cortex (1240/1658 cm^–1^) comparing to the appropriate control group.

#### Relative Surfaces of the Internal Capsule Region

**Figure 10:**
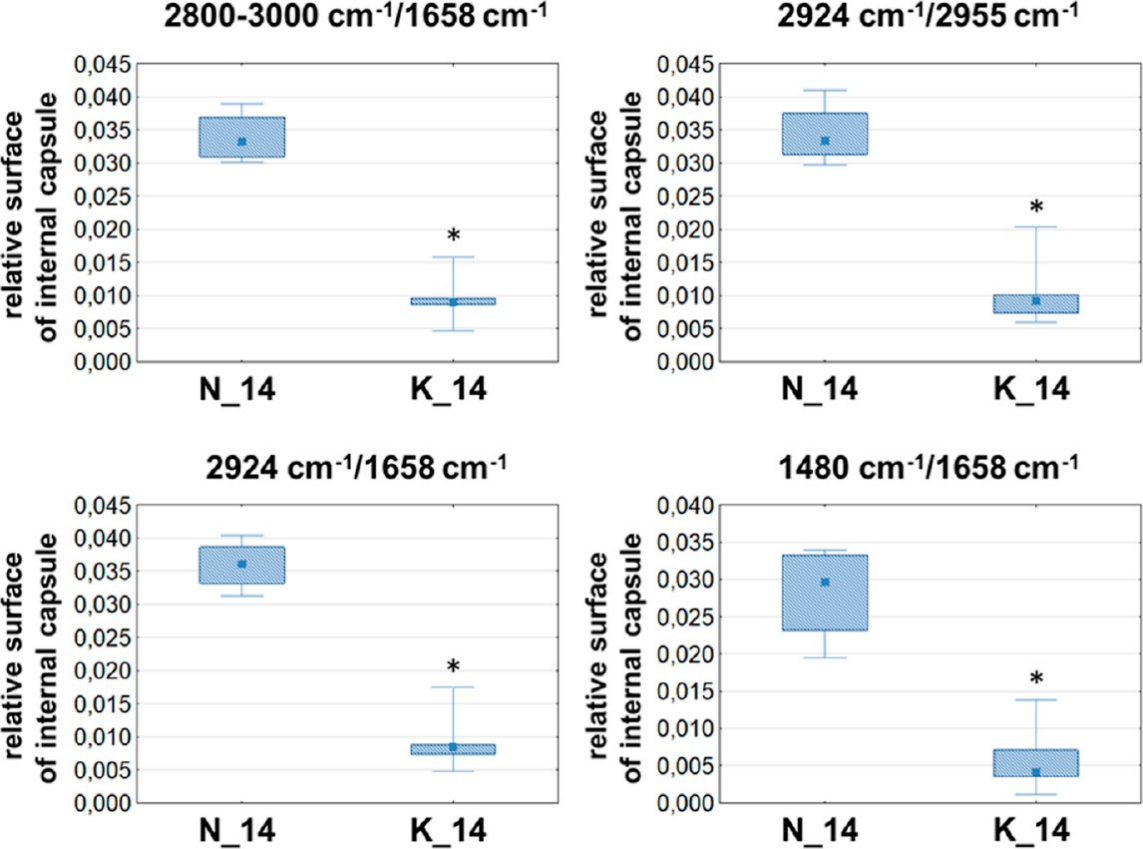
Box-and-whisker plots presenting the spread of the relative surface of the internal capsule for 14 days old experimental and control rats (K and N groups, respectively). Statistically significant differences (Mann–Whitney *U* test, 95% confidence level) between groups were marked with *.

The results of chemical mapping performed for lipid bands showed that the surface of internal capsule (structure of the white matter) is smaller for the 14 days old offspring of females fed with KD than with normal one. To confirm this result the relative, comparing to the whole brain slice, surface of the area was determined for each animal. This was done on the basis of the chemical maps presenting the following biochemical parameters: 2800–3000/1658, 2924/1658, 2924/2955, and 1480/1658 cm^–1^. To estimate the surface of the internal capsule and whole brain slice, ImageJ software (version 1.52a) was applied. The calculated relative surfaces were subjected to statistical evaluations with the use of the Mann–Whitney *U* test to verify the relevance of the differences between experimental animals and the appropriate controls. As one can see from Figure 10, the performed statistical analysis confirmed the prior qualitative observations done on the basis of the chemical mapping results.

## Discussion

In the present study, FTIR microspectroscopy was, for the first time, used to assess biochemical changes, which may occur in the nervous system of the offspring as an effect of maternal KD. The chemical mapping was performed on the whole brain slices which included the dorsal part of the hippocampal formation.

Analysis of chemical maps obtained for 2 and 6 days old animals, mothers of which were fed with normal or KD during pregnancy, generally did not show the differences in the accumulation and structure of examined biomolecules. The only exception from this observation concerned the ratio of 1080/1658 cm^–1^ bands intensity for 2 days old rats. Namely, the mentioned biochemical parameter presented higher values in case of the offspring of females fed during pregnancy with ketogenic chow. PCA did not show the relevance of the differences between the spectra recorded within the examined areas and cellular layers for experimental animals and the appropriate controls. Nonetheless, further semi-quantitative biochemical analysis and the results of Mann–Whitney *U* test confirmed the increase of the relative intensity of 1080 cm^–1^ absorption band for corpus callosum in case of 2 days old rats exposed prenatally to KD. What is more, a similar relation was detected for the relative intensity of the band at 1240 cm^–1^ within the brain cortex. The intensity of the bands at 1240 and 1080 cm^–1^ is related to the accumulation of the compounds containing phosphate groups, including nucleic acids, phospholipids, and may be the source of the information about the differences in the degree of phosphorylation of carbohydrates or glycoproteins.^35−37^ The band at 1080 cm^–1^ is characteristic also for carbohydrates, and the changes in its intensity may reflect the fluctuations in the tissue glucose levels. The females fed during pregnancy with KD, on the 2nd day after delivery started to obtain standard chow which significantly increased the availability of carbohydrates for them. The change of mother diet, however, does not seem to be able to elevate immediately the brain glucose levels of the offspring. Especially, that maternal milk is always rich in fats and the transport capacity of glucose through the blood–brain barrier is poor at the beginning of the postnatal life.^38^ Later, glucose availability in pups organism is progressively increasing due to both the increased expression of cerebral glucose transporters and the intensified activity of glycolytic enzymes. The final “adult” levels of glucose metabolic rates are achieved on the 30th day of postnatal life.^39^

Chemical mapping showed general elevated relative level of 1740 cm^–1^ band intensity for 14 days old rats from K group. The detailed semi-quantitative and statistical analysis confirmed these qualitative observations for cortex and two cellular layers of hippocampal formation (multiform and molecular). Because the levels of proteins did not differ between the experimental and control groups, the observed changes are associated with the increased accumulation of compounds containing carbonyl groups, such as phospholipids, cholesterol esters, and ketone bodies. Although lipids themselves have difficulties crossing the placenta, they can, indirectly, simplify the transport of other substances to the fetus.^40^ During KD, free fatty acids present in the blood are carried to the liver where, in the process of β-oxidation, are degraded what leads to the production of KB: acetoacetate, acetone, and β-hydroxybutyrate.^41−44^ All KB can more easily, comparing to glucose, cross the blood–brain barrier and therefore, they may be observed in the nervous tissue of KD fed animals.^42,44^ The literature evidence indicates, moreover, that KB circulating in the maternal blood can easily cross the placenta and reach the same levels as those in maternal plasma.^40^ The immature brain is able to take up KB from two to three times more efficiently than the mature one and use them for energy metabolism and lipid as well amino acid biosynthesis.^38,45−47^ Our earlier study carried out on adults male Wistar rats showed increased intensity of the band at 1740 cm^–1^ in hippocampal formation of animals fed with KD.^42^ On the other hand, we did not notice an elevated level of this band in the females fed with KD 2 days postpartum, although the level of β-hydroxybutyrate in their blood was significantly higher both on the day of fertilization as well as on 4th, 15th, and 20th gestational day.^10^

Some crucial observations have been done for the area of white matter of 14 days old offspring of rats fed prenatally with KD. The qualitative analysis of chemical maps showing the relative content of lipids (2800–3000/1658, 2924/1658 cm^–1^), cholesterol, and its esters (1480/1658 cm^–1^), as well as the changes in the lipid structure (2924/2955 cm^–1^), revealed smaller surface of the internal capsule area in the case of the offspring of KD-fed females. The relevance of the mentioned effect was confirmed by the further semi-quantitative biochemical analysis and the results of the Mann–Whitney *U* test, which showed also lower relative content of lipids and their structural abnormalities for other white matter area, namely, corpus callosum. The results obtained are quite surprising taking into account the fact that lipids are the main energy source during KD. What is more, the accumulation of fatty acids in the brain of animals from the K group should be greater, with the elevated level of the 1740 cm^–1^. This absorption band is associated with KB, which are the precursors for the synthesis of lipids (mainly cholesterol) and amino acids, especially in the neonatal period.^48^ They also constitute the substrates in the myelination process.^48^ Therefore, one might expect that their elevated availability should rather lead to the increased area of the structures of the white matter. On the other hand, cerebral KB metabolism is regulated by the permeability of the blood–brain barrier (BBB), which in rats, increases during the suckling term, even if mothers did not obtain special, high-fat diet during pregnancy.^48^ According to the study of Gjedde and Crone (1975), the BBB contains a transporter for short-chain monocarboxylic acids, such as KB and lactates.^49^ Shortly after birth, the brain temporarily uses lactate as a source of metabolic substrates, followed by the suckling period in which metabolism is dependent mostly on ketones.^39^ During lactation, the offspring has higher circulating ketone levels, elevated amount of the BBB transporters, and greater enzymatic activities of some ketone metabolizing enzymes.^39^ In rat brain, changes in the three mitochondrial enzymes of KB utilization (3-hydroxybutyrate dehydrogenase, succinyl-CoA 3-oxoacid CoA transferase, and mitochondrial acetoacetyl-CoA thiolase) associated to the age are present.^48,50^ Activity of these enzymes is relatively low at birth but steadily elevates through the lactation to a maximum at the time of weaning—about 21 days from delivery.^48,50^ Reassuming, the changes in these enzyme activity are simultaneous to the changes in BBB permeability for KB and they together enable the brain to use the high levels of KB circulating in the blood during the suckling period.^48^ In turn, the cytoplasmic enzymes of cerebral KB metabolism—acetoacetyl-CoA synthetase and cytoplasmic acetoacetyl-CoA thiolase—show the opposite changes in the activity with the development. The activity of both enzymes is the highest at birth and falls gradually to 25–50% of the initial value in adult rats.^48,50,51^ The mentioned cytoplasmic enzymes are used for the synthesis of lipids, particularly cholesterol, which is necessary for the process of myelination.^48^ Myelination starts in rat at birth and lowers considerably by the age of 30 days, correlating with the changes in the cytoplasmic enzymes activity.^48^

In the case of females, which additionally receive KD during feeding their offspring, the mentioned relations may be intensified and lead to metabolic acidosis. This may connect not only with the developmental brain disorders leading to worse performance of neurodevelopmental reflexes but also with the body mass reduction and the delayed muscle development.^10,18^ In our study, the mothers of rats from K groups were fed with KD only during gestation and then since 2nd day postpartum, during suckling term, they obtained the normal diet. Taking into account this fact and the above-presented considerations, our outcomes may testify about the process of compensation occurring in mothers organisms. When they started to obtain the normal diet with the standard amount of carbohydrates, their organisms slowly began to replenish with the deficiencies of these compounds, leaving KB to the young rats as the main source of the energy for the metabolism.

It is also worth mentioning here, that at the beginning of postnatal life, the offspring of mothers fed with KD during pregnancy had significantly lower body mass what was described in details in our previous work.^10^ When, on the 2nd day postpartum, in KD-fed mothers, normal diet was introduced, and their progeny began to rebuilt the body mass very quickly. Although on the 6th day of postnatal life body mass was still significantly lower comparing to control group, the differences between the experimental and control group were much less pronounced. On the 14th day after birth, the offspring showed complete restoration of their body mass.^10^ This is particularly interesting considering the fact that we have noticed an inverse relation in the biochemical changes in the brains of the experimental animals: almost none of the abnormalities were detected in 2 and 6 days old animals that were just regaining body weight, while some differences were evident in the 14 days old rats.

Taking into account the developmental changes in the relative intensity of the 1080 cm^–1^ band, it was the highest on the 6th day of postnatal life for both experimental and control animals with no significant differences between them. Also the relative intensity of the band at 1740 cm^–1^ changed during postnatal development but it gradually increased until the 14th day of animal life. Reassuming, the cerebral accumulation of the carbohydrates and KB changes together with the brain development and depends on the diet of mother during pregnancy. This study should be continued. Especially, the adult offspring of KD-treated pregnant females should be examined to verify if observed biochemical alterations are of temporary or permanent nature.

## Limitations of the Study

This study and conclusions resulting from it have some limitations, which we would like to discuss in this chapter.

### Interpretation of Measured Biochemical Parameters

FTIR microspectroscopy is based on the absorption of IR radiation by the vibrating molecules. The functional groups are the parts of the molecules that determine their major properties. Each functional group has its own discrete vibrational energy which depends on the atoms present in it, type, and strength of bonds. The vibrations are unique to particular functional groups and, therefore, can be used to identify molecules. FTIR microspectroscopy provides information on a wide range of molecular classes, such as lipids, proteins, carbohydrates, and others. Although these biomolecules have individual spectral signatures, which are called “finger prints”, absorption bands tend to overlap if the biomolecules have common molecular vibrational modes. Because of that, the use of integrated bands or their ratios to estimate the content and structural anomalies of biomolecules in tissues is complicated. As such analysis indirectly inform about the presence and distribution of biological macromolecules, it is called “semi-quantitative”. The tissues may be also very complex and heterogeneous which causes that the interpretation of their IR absorption spectra may be in some cases ambiguous.

The interpretation of tissue IR spectra in this study was done according to the established knowledge based on many previously published papers confirming the usefulness of FTIR microscopy for the biomolecular analysis of such type of samples. According to the work of Kneipp et al., the band at 1600–1700 cm^–1^, called amide I band, provides information about proteins accumulation and, what is more, it is their conformation-sensitive.^52^ Other studies have reported that vibrational frequencies characteristic for α-helices and β-sheets occur, respectively, at the wavenumbers of approximately 1655 and 1630 cm^–1^ and, therefore, the ratio of the absorbance at these wavenumbers may provide information about structural changes/abnormalities of proteins.^53,54^ In turn, the spectral range of 2800–3000 cm^–1^ is dominated by the absorption bands connected with the asymmetric and symmetric C–H stretching vibrations of the CH_2_ and CH_3_ groups of fatty acids.^52,55−57^ The compounds containing carbonyl groups may be recognized in the IR spectrum by the band found at the wavenumber of 1740 cm^–1^ and characterisic for C=O stretching vibrations.^58,59^ At lower wavenumbers (1000–1300 cm^–1^) spectrum is dominated by P=O stretching vibrational modes of compounds containing phosphate groups such as phospholipids and nucleic acids and also characteristic vibrations of DNA/RNA backbone and carbohydrate structures (C–O stretching vibrations).^33,52,54,60^ The most complex spectral region is the wavenumber range of 1360–1480 cm^–1^, which contains information about the presence of fatty acids, cholesterol, and its esters.^33,60^ The tentative assignment of the vibrational modes and biomolecules to the band frequencies present in the IR spectra measured in nervous tissue are shown in Tables 2 and 3.

### Small Number of Animals Used in the Study

Another limitation of this study is small number of animal used in the experiment (five rats per group). Scientific research should be carried out in accordance to 3Rs which provides a clear set of directions for improving the welfare of experimental animals and enables improving scientific results.^61^ Especially important are rules associated with the minimization of animals suffering and reduction in the number of animals used to obtain comparable level of information as in the larger groups. In such a case, however, the use of parametric tests that require normal distribution of variables in population is not recommended. That is why non-parametric Mann–Whitney *U* test was applied in the study to verify the statistical significance of the differences between experimental groups and appropriate controls.

### Statistical Analysis

The Mann–Whitney *U* test with 95% confidence interval was applied here to verify the statistical significance of biomolecular and morphological differences between experimental groups and appropriate controls. The choice of the nonparametric statistical test was dictated by the fact that our data might have not fulfill the assumptions about normality, homoscedasticity and linearity, which are necessary for the use of parametric one. The Mann–Whitney *U* test is the most commonly used alternative for the two-sample Student *t*-test. The typically used significance levels for the *U* test, are analogical as in case of parametric tests, namely, 0.01, 0.05, or 0.1.

Perme and Manevski showed the alternative to the Mann–Whitney *U* test based on the degree of the variable distributions overlap and proposed several algorithms for the construction of the confidence interval for this method including the Newcombe’s 5th or DeLong method.^62^ Although both algorithms seem promising, they are based on a parametric approach to variance estimation and, what is more, may be inadequate in case of small sample sizes. Another problem is that this attitude is still rarely used for reporting the confidence intervals and it would be difficult to compare our results with the experimental results of other research groups.

## Conclusions

The results presented in this study confirmed the usefulness of FTIR microspectroscopy in the investigation of the influence of KD used during pregnancy on the biochemical status of the nervous system of the offspring. The topographic analysis of chemical maps showed that the distribution and accumulation of biomolecules in brains of young rats depend both on the stage of postnatal development and the female diet during pregnancy. The offspring of females subjected to KD presented the differences in the relative levels of the bands at 1080 and 1740 cm^–1^ which point at the alterations in the distribution of KB and glucose in their brains. What is more, it was stated that the KD treatment during fetus life may lead to the decrease in the size of internal capsule of progeny and the changes in the relative level and structure of lipids in white matter. FTIR microspectroscopy turned out to be extremely helpful in assessing the biochemical composition of the tested samples. However, new light on the obtained results could be shed by the data concerning the elemental anomalies occurring in the changed tissue areas as well as more detailed, carried out with better spatial resolution, and biomolecular analysis.

## Materials and Methods

### Animals

The animal husbandry and all procedures associated with them were carried out in the Department of Experimental Neuropathology (Institute of Zoology and Biomedical Research of Jagiellonian University) in accordance with the permission no. 122/2015 of the First Local Ethical Committee and with the international standards. In the experiment, the offspring (at the age: 2, 6, and 14 days of the postnatal life) of the rats fed with the ketogenic or standard diet during pregnancy were used. To control fertilization, 2 month old female Wistar rats were placed in one cage with males during the night and in the morning, those sperm-positive females were set in a separate cages for the period of pregnancy. Pregnant rats were randomly divided into two groups. The first one remained on the standard laboratory diet whilst the second was fed with KD. Both diets were continued during the whole gestation. In turn, during the lactation period both groups obtained standard laboratory diet which, in the case of the previously KD fed females, was introduced 2 days after labor. The pregnant rats had the access to food and water ad libitum, and the mass of the consumed chow was controlled three times a week. Once a week, the females were weighed and the levels of the ketone bodies and glucose were measured in their blood.

Postpartum, the gender of the offspring was identified and afterward the animals of each sex were randomly divided into groups differing in the times of the perfusion and the collection of samples. The number of rats was five per each experimental group. The brains were taken from them on the 2nd, 6th, and 14th day of life, and the chosen periods correspond to the key processes occurring in the rat brain during its postnatal development.

### Ketogenic and Standard Laboratory Diet

In the study, we used KD with long-chain fatty acids (ssniff EF R/M with 80% Fat) and the standard laboratory diet in the form of Labofeed (Morawski). The selected KD is characterized with high ketogenic ratio (KR), which is the mass ratio of fats to proteins and carbohydrates.^63^ The choice of a high KR diet is related to the fact, that it is usually more efficient in the seizure control.^64,65^ The comparison of the content of main nutrients in the dry mass of the used ketogenic and standard chow is done in Table 1.

**Table 1:** Content of Main Nutrients (% of the Dry Mass) in the Ketogenic and Standards Laboratory Diet

nutrient	KD	standard diet
lipids	79	10
carbohydrates	1	60
proteins	8	30
others	12	0

### Sample Preparation

On the 2, 6, or 14 days of postnatal life rats were deeply anesthetized with Morbital (Biowet) and perfused with physiological saline solution of high analytical quality. The brains were excised and deeply frozen in liquid nitrogen. Twelve micrometer thick slices with the dorsal part of the hippocampal formation were cut using cryomicrotome and placed on sample carriers made of CaF_2_, and dedicated to measurements with FTIR microspectroscopy in the transmission mode.

### IR Data Collection

The biochemical analysis of brain samples was performed using FTIR microspectroscopy. The measurements were performed at the Faculty of Physics and Applied Computer Science of the AGH University of Science and Technology (Krakow, Poland). Thermo Scientific Nicolet iN10 MX infrared microscope, equipped with a ceramic radiation source, was used for the study. For faster scanning of samples and chemical imaging, the ultrafast mapping system and a linear array of mercury cadmium telluride (MCT) detectors were used. In turn, the single spectra from areas of interest were recorded with the point MCT detector. The samples deposited on CaF_2_ slides were analyzed in transmission mode with a spatial resolution of around 25 μm. The spectra were recorded for the wavenumber range 4000–900 cm^–1^ with spectral resolution set to 8 cm^–1^. 32 scans were averaged per both sample and background spectrum. The data acquisition as well as spectral analysis were performed with OMNIC Picta software (version 8.1).

### Topographic and Semi-Quantitative Biochemical Analysis, Statistical Evaluation of the Results

The topographic analysis of the main biological macromolecules in the brain was based on the chemical mapping of their absorption bands or the ratios of their absorption bands. For this purpose OMNIC Picta software (version 8.1) was used. The two-dimensional chemical maps were generated by imaging of the area of one peak or the area ratio of two peaks, including trapezoidal baseline correction. In one case, for the wavenumber region between 2800 and 3000 cm^–1^, we did not examine the intensity of the bands, but the integrated absorbance within this particular wavenumber range. The mentioned spectral range, called lipid massif, includes a few absorption bands specific to lipids. Also, in this case, trapezoidal baseline correction was applied. In Table 2, the tentative assignments of the band frequencies characteristic for IR spectra measured in nervous tissue are shown. In turn, the characteristics of the absorption bands and the ratios of absorption bands examined in this study are presented in Table 3.

**Table 2:** Tentative Assignments of the Band Frequencies Characteristic for IR Spectra Measured in the Nervous Tissue^42,52−54,60,66^

frequency [cm^–1^]	assignment
∼2954	CH_3_ asymmetric stretching (saturated fatty acids)
∼2924	CH_2_ asymmetric stretching (saturated fatty acids)
∼2870	CH_3_ symmetric stretching (saturated fatty acids)
∼2852	CH_2_ symmetric stretching (saturated fatty acids)
∼1730	C=O stretching (phospholipids, cholesterol ester)
∼1640–1653	C=O stretching, C–N stretching, N–H bending (proteins, sphingolipids)
∼1545–1567	N–H bending, C–N stretching (proteins, sphingolipids)
∼1460–1473	CH_2_ scissoring, CH_3_ asymmetric bending (fatty acids)
∼1443	CH_2_ (cyclic) scissoring (cholesterol, cholesterol ester)
∼1378–1381	CH_3_ symmetric bending (fatty acids)
∼1365	CH_2_ symmetric bending (fatty acids)
∼1200–1400	C–N stretching, N–H bending, C=O stretching, O=C–N bending (proteins)
∼1228–1244	PO_2_ asymmetric stretching (nucleic acids, phospholipids)
∼1170	CO–O–C asymmetric stretching (phospholipids)
∼1084–1089	PO_2_ symmetric stretching (nucleic acids, phospholipids)

**Table 3:** Examined Biochemical Parameters^67,68^

absorption band/ratio of absorption bands	biochemical parameter
1658 cm^–1^	amide I band, distribution of proteins
1635/1658 cm^–1^	structural changes of proteins (β-sheet to α-helix ratio)
2924/2955 cm^–1^	structural changes of lipids
2800–3000/1658 cm^–1^	distribution of lipids (lipid massif) in relation to proteins
2924/1658 cm^–1^	distribution of lipids in relation to proteins
1240/1658 cm^–1^	distribution of compounds containing phosphate groups in relation to proteins
1080/1658 cm^–1^	distribution of compounds containing phosphate groups in relation to proteins
1740/1658 cm^–1^	distribution of compounds containing carbonyl groups in relation to proteins
1360/1658 cm^–1^	distribution of lipids, cholesterol and its esters in relation to proteins
1480/1658 cm^–1^	distribution of lipids, cholesterol and its esters in relation to proteins

**Figure 11:**
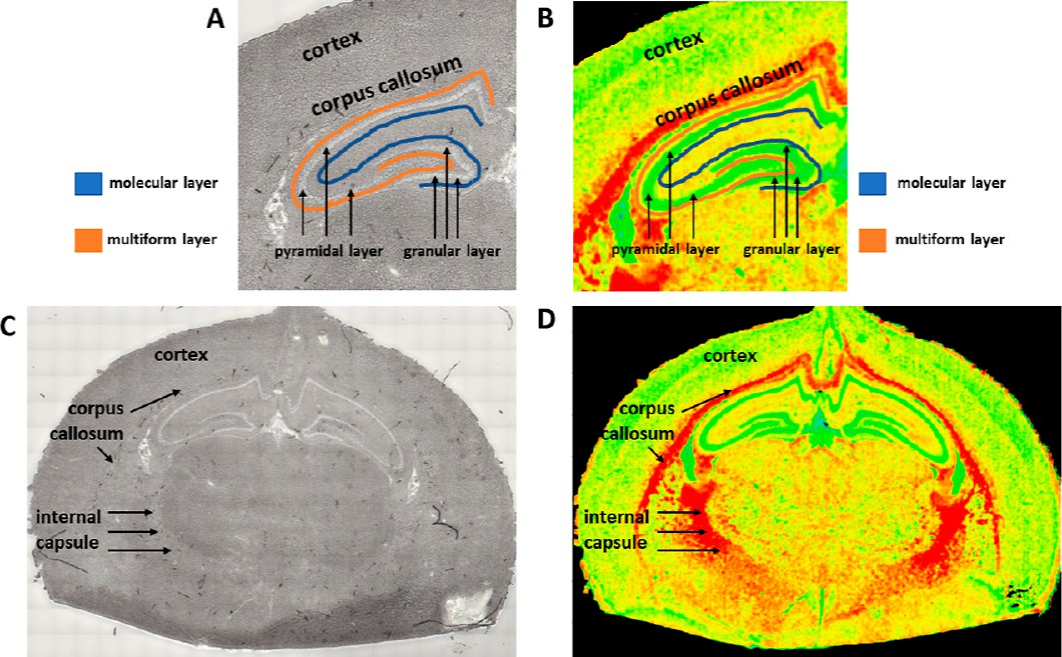
Localization of the areas (two structures of white matter, namely, corpus callosum and internal capsule, and cortex) and cellular layers (granular, pyramidal molecular, and multiform layer) of interest, presented in the microscopic images (A,C) and chemical maps (B,D) showing the distribution of lipids for the selected sample of the brain.

For the statistical evaluation of the spectral data and identification of the potential differences between experimental and control groups, the principal component analysis (PCA) and Mann–Whitney *U* test were utilized. At first, important brain regions including brain cortex, corpus callosum (structure of the white matter), and four cellular layers of hippocampal formation, namely, granular, pyramidal, multiform and molecular, were localized in each examined tissue slice (Figure 11). Then, with the use of point MCT detector, 100 spectra in the randomly chosen points from the mentioned areas of interest were collected for each animal. For the purposes of PCA of the spectral data the Origin Pro software (version 2020b) was applied. The preprocessing of the spectra included atmospheric and baseline correction as well as vector normalization. The PCA was done based on the second derivative spectra.

In the next step, for each animal and examined area/cellular layer, the absolute and/or relative average intensities of chosen absorption bands (given in Table 3) were calculated. Afterward, the non-parametric Mann–Whitney *U* test was applied to verify the statistical significance of the differences in the obtained biochemical parameters between animals exposed prenatally to KD and the controls at the appropriate stage of postnatal development. For statistical analysis, STATISTICA software (version 14.0.1.25) was used, and the significance level was assumed as 5%. The Mann–Whitney *U* test was also applied in order to confirm the statistical significance of the differences in the relative surface of the internal capsule between experimental groups and appropriate controls.

## Supporting Information Available

Microscopic views of the scanned brain tissue areas of 2, 6, and 14 days old animals (PDF)
