# Supplementary material for: Does Ketogenic Diet Used in Pregnancy Affect the Nervous System Development in Offspring?—FTIR Microspectroscopy Study
Source: ACS Chem Neurosci. 2023 Jul 20;14(15):2775–91. doi: 10.1021/acschemneuro.3c00331 (PMC10401638; doi:10.1021/acschemneuro.3c00331)
Supplement: Supplementary file 1 — cn3c00331_si_001.pdf [file cn3c00331_si_001.pdf]

Supporting Information

DOES KETOGENIC DIET USED IN PREGNANCY AFFECT THE NERVOUS SYSTEM DEVELOPMENT IN OFFSPRING? – FTIR MICROSPECTROSCOPY STUDY

Marzena Rugiel[1#], Zuzanna Setkowicz-Janeczko[2#], Wojciech Kosiek[2], Zuzanna Rauk[2], Kamil Kawon[1], Joanna Chwiej[1*]

[1]*Faculty of Physics and Applied Computer Science, AGH University of Krakow, Krakow 30-059, Poland*
[2]*Institute of Zoology and Biomedical Research, Jagiellonian University, Krakow 31-007, Poland*

# equally contributed

* Corresponding author; email address: joanna.chwiej@fis.agh.edu.pl

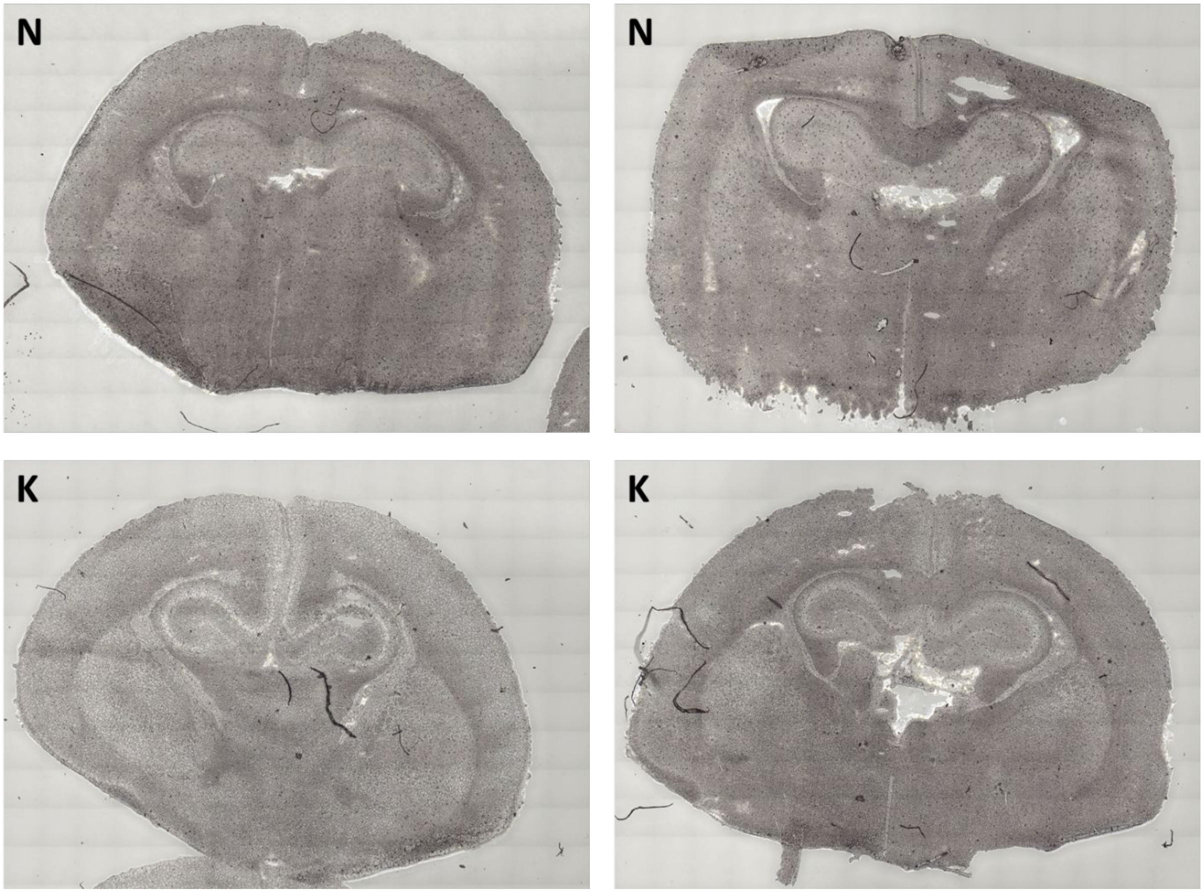

Figure S1. The microscopic views of the scanned brain tissue areas of 2-days old animals.

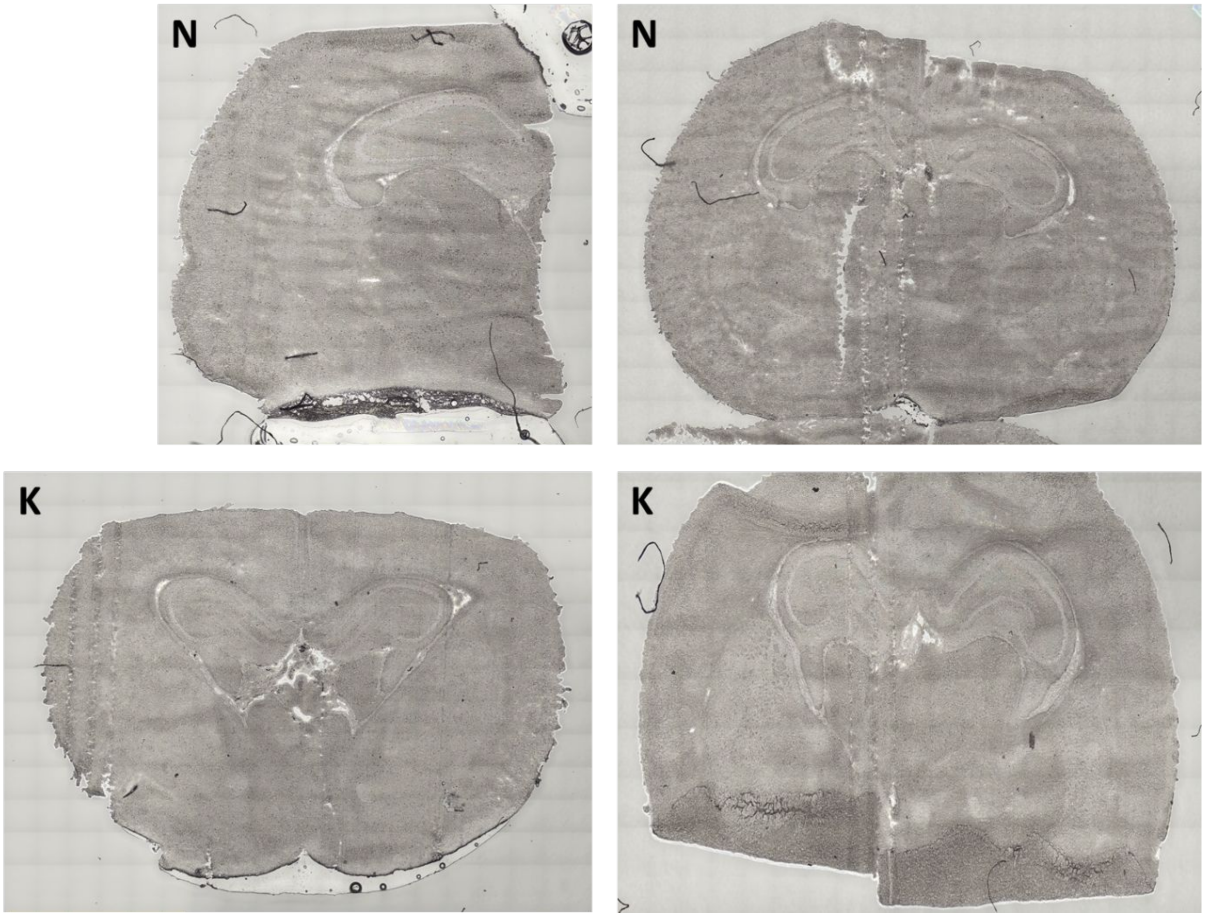

Figure S2. The microscopic views of the scanned brain tissue areas of 6-days old animals.

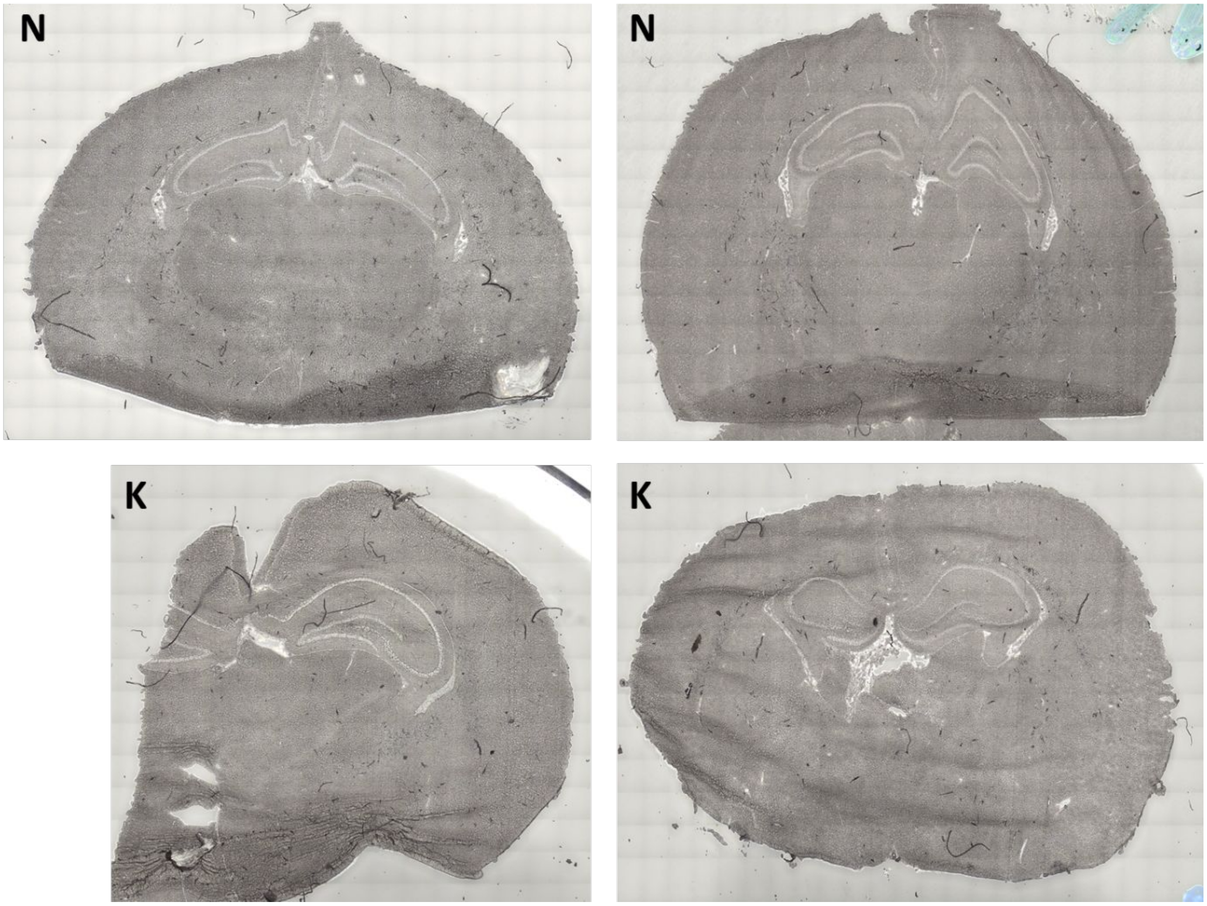

Figure S3. The microscopic views of the scanned brain tissue areas of 14-days old animals.
